# Evaluation of analytical performance of the Revvity sFlt-1 and PlGF methods for the assessment of preeclampsia

**DOI:** 10.3389/bjbs.2026.15950

**Published:** 2026-07-15

**Authors:** Rhiannon Marr, Ana Sofia Cerdeira, Ian Smith, Manu Vatish, Tim James

**Affiliations:** 1 Department of Clinical Biochemistry, Oxford University Hospitals NHS Foundation Trust, Oxford, United Kingdom; 2 Nuffield Department of Women’s and Reproductive Health, University of Oxford, Oxford, United Kingdom

**Keywords:** angiogenic markers, placental growth factor, preeclampsia, pregnancy, soluble fms-like tyrosine kinase 1

## Abstract

**Introduction:**

The measurement of the angiogenic biomarkers placental growth factor (PlGF) and soluble fms-like tyrosine kinase-1 (sFlt-1) are increasingly used to support the prediction and diagnosis of preeclampsia (PE) in routine clinical practice. There are an increasing number of methods available for the analysis of these markers but data showing their analytical comparability is limited.

**Materials and methods:**

The assays for PlGF and sFlt-1 from Revvity were evaluated and compared against Roche methods that are used in current clinical practice in Oxford. Imprecision and paired analytical comparisons studies were undertaken and data evaluated for numeric agreement and concordance relative to manufacturer recommended rule-in and rule-out thresholds for PE.

**Results:**

Imprecision estimates for the Revvity PlGF and sFlt-1 methods calculated from quality control material analysed during the evaluation were between 3.2 and 9.0 CV%. Revvity method precision profiles derived from 581 clinical specimens analysed in duplicate had a median CV% for PlGF of 1.8%, IQR 2.3% and for sFlt-1 a median CV% of 1.1%, IQR 1.5%. Comparison against the Roche PlGF and sFlt-1 methods in 437 clinical specimens showed an overall Passing-Bablok regression relationship for PlGF of y = −23.4 + 0.73x (r = 0.983) and for sFlt-1, y = −87.7 + 0.40x (r = 0.971). However, there was statistically significant (*p < 0.0001*) concentration dependant relative bias for both methods and the calculated ratio. Concordance of the sFlt-1:PlGF ratio relative to manufacturer specific rule-in and rule-out thresholds was 95.2%.

**Discussion:**

The Revvity methods for PlGF and sFlt-1 are precise and correlate with the Roche methods. However, numeric agreement precludes result interchangeability and the use of common rule-in and rule-out thresholds. The manufacturer specific thresholds should be applied in clinical practice. Further work is required to understand how method differences impact clinical outcomes and their causes.

## Introduction

Preeclampsia (PE) is a multisystem disorder of pregnancy presenting after 20 weeks’ gestation which is estimated to affect between 2% and 5% of women in developed countries [1]. The condition can have serious complications, which include premature delivery, neonatal mortality and maternal death [2]. PE is defined by hypertension and proteinuria, or end organ dysfunction [3]. Whilst hypertension and proteinuria are hallmarks of preeclampsia, they are non-specific findings during pregnancy, and it is not possible to accurately predict disease using these signs alone [4].

Pathophysiological studies of PE have identified that the development of the condition is associated with a disturbance of factors associated with angiogenesis [5]. Specifically, the antiangiogenic protein soluble fms-like tyrosine kinase-1 (sFlt-1) is elevated, and the pro-angiogenic protein placental growth factor (PlGF) is reduced in the circulation of pregnant mothers with PE compared to those who do not have PE. Consequently, these proteins have emerged as diagnostically useful markers that can improve management of the condition when patients present with symptoms [6]. Clinical studies have consistently demonstrated that measurement of these markers has benefit for patient care, in different countries and health systems, and using a growing number of manufacturers’ automated immunoassays [7–11].

In May 2016 the National Institute for Health and Care Excellence (NICE) published its first guidance on the use of PlGF based testing in suspected PE, diagnostic guidance DG23 which supported the use of these markers as rule-out tests [12]. The specific tests supported were the Triage PlGF test and the Roche Elecsys immunoassay sFlt-1/PlGF ratio. Following the release of NICE guidance, the adoption of the markers into UK routine clinical practice was slow, and several barriers to implementation were suggested, including cost uncertainty, benefits realisation, and awareness [13]. Following review of additional evidence, NICE diagnostic guidance DG49 was published in July 2022 and recently reissued, without amendment, as HealthTech guidance HTG630 [14]. The expanded guidance included use of PlGF based testing as a rule-in test, and inclusion of the methods from PerkinElmer (now Revvity) – the DELFIA Xpress PLGF 1-2-3 test and the DELFIA Xpress sFlt-1/PLGF 1-2-3 ratio.

The performance of different methods of measurement for the angiogenic markers have been compared, both analytically and clinically [15–20]. These studies show considerable quantitative differences in numerical results for both PlGF and sFlt-1 which lead to method dependent rule-in and rule-out clinical thresholds for PE [14]. Factors that may influence the degree of result concordance between methods include calibration materials, calibration preparation, variable isoform cross-reactivity and the characteristics of the patient population tested, such as gestational age, ethnicity and clinical conditions.

In the present study we evaluate the analytical performance of the DELFIA Xpress sFlt-1 and PlGF 1-2-3 assays, including a comparison against the Roche Elecsys methods, in a large population requiring testing for suspected preeclampsia against the NICE guidance [14].

## Materials and methods

### Patient specimens

The samples used in the present study were those routinely collected for clinical care against NICE guidance HTG630 [14]. Blood was collected into Becton-Dickinson SST-II advance serum tubes (Becton Dickinson, Wokingham, UK), centrifuged and analysed on the Roche assay, usually within 2–3 h of collection. Where immediate analysis was not possible, the centrifuged samples were stored refrigerated at 2 to 8 °C for up to a maximum of 16 h, before analysis. Testing for PlGF and sFlt-1 on the Revvity analysers was undertaken as a service evaluation and registered on the hospitals clinical governance audit system (reference ID 7086).

Following analysis using the Roche method to provide the result for clinical management, specimens were aliquoted and frozen at −80 °C. These samples were thawed at room temperature and mixed by gentle inversion 10-15 times and analysed on the Revvity method. To assess the potential impact of the freeze-thaw cycle a second group of samples were analysed concurrently on both the Roche e411 and DELFIA® Xpress analysers for both PlGF and sFlt-1. These two subsets allowed comparison of method agreement for unfrozen samples, analysed with a workflow reflective of clinical practice, relative to samples stored frozen.

Patients who had more than one specimen tested were only included once in the assessment of the Revvity methods comparability to the Roche methods. As the comparison between methods focused on the use of the markers for suspected PE [14] those with twin pregnancies, and those tested at gestational ages outside the current guidance of 20–36+6 weeks’ gestation [14] were excluded.

### Laboratory methods

The Revvity (formerly PerkinElmer) assays evaluated were the PlGF 1-2-3™ kit (Ref: 6007-0050) and sFlt-1 kit (Ref: 6009-0010) applied on the DELFIA® Xpress (Wallac Oy, Turku, Finland) and used as recommended by the manufacturer. Limits of quantitation (LoQ) and limits of detection (LoD) for sFlt-1 and PlGF Revvity assays are 7.6 pg/mL and 3.8 pg/mL, and 3.3 pg/mL and 1.9 pg/mL, respectively. Measuring ranges were 0.4–28,900 pg/mL sFlt-1and 1.9–5650 pg/mL for PlGF.

The comparator methods were those used for routine laboratory service within the Clinical Biochemistry at Oxford University Hospitals NHS Foundation Trust, the Elecsys sFlt-1 (Ref: 05109523190) and Elecsys PlGF (Ref: 05144671190) kits analysed on the Roche Elecsys® cobas e411 (Roche Diagnostics, Burgess Hill, UK) according to manufacturer’s guidance. LoQ and LoD for sFlt-1 and PlGF Roche assays are 15 pg/mL and 10 pg/mL, and 10 pg/mL and 3 pg/mL, respectively. Measuring ranges were 10–85,000 pg/mL for sFlt-1and 3–10,000 pg/mL for PlGF. Both methods are within the laboratories UKAS accredited scope of testing and enrolled in an EQA scheme with satisfactory performance. Intermediate imprecision calculated from internal quality control data for the Roche PlGF method was 3.0% at 106.1 pg/mL and 2.9% at 1,068.5 pg/mL and for the Roche sFlt-1 method was 2.0% at 103.6 pg/mL and 2.4% at 1,007.8 pg/mL.

### Method calibration

The Roche Elecsys instructions for use (IFU) (Ref: 05144671500v12.0 and 07027818500v6.0 for PlGF and sFlt-1 respectively) state Roche calibrators for both PlGF and sFlt-1 are traceable to commercially available sFlt-1 and PlGF ELISA assays. The methods utilise a master calibration curve that is established during manufacturing and is calibrator lot specific. At each laboratory site the calibration characteristics are read from a calibrator barcode and a two-point calibration run to adjust the stored master calibration curve [21, 22]. The Revvity assays were calibrated using a series of six calibrants (A–F) of increasing concentration from zero to 4,000 pg/mL for PlGF, and from zero to 19,500 pg/mL for sFlt-1 which were assigned values based on manufacturers in-house primary calibrators. For PlGF the primary calibrator was prepared with recombinant human PlGF the value of which was determined with a RP-HPLC method which used D-tryptophan as an internal standard. For sFlt-1 the primary calibrator series was prepared with recombinant human sFlt-1 the value of which was determined with amino acid analysis performed by HPLC after acid hydrolysis, using sarcosine as an internal standard.

### Assessment of imprecision

Intermediate imprecision was assessed using the two Revvity kit IQC materials provided and analysed in each analytical batch undertaken; Revvity, PLGF controls (Ref: 3090-0010) lot 252, and Revvity sFlt-1 controls (Ref: 3246-0010) lot 853. To assess the imprecision over the wider concentration seen in clinical practice duplicate analysis of patient samples was undertaken and used to construct a precision profile. Imprecision is expressed a percentage coefficient of variation (%CV).

### Clinical categorisation against manufacturer recommended thresholds

The sFlt-1:PlGF thresholds recommended by manufacturers to help diagnose PE differ with respect to intended use and gestational age [14]. The sFlt-1:PlGF ratios were defined as either rule-in, rule-out, or intermediate if the result was between the respective manufacturer’s gestation specific rule-in and rule-out thresholds. For the rule out of PE between 20 and 37 weeks using the Roche method a ratio ≤38 was used whereas for the Revvity method a ratio ≤50 was used. For GA of 20–34 weeks, the rule in of PE using the Roche method a ratio ≥85 was used compared to a ratio ≥70 was used for the Revvity method. Intermediate risk results for the Roche method were those ratios above 38 and below 85 and for Revvity above 50 but below 70. For GA of ≥34 weeks, the rule in of PE using the Roche method a ratio ≥110 was used, compared to a ratio of ≥90 for the Revvity method.

### External quality assessment

At the time of the evaluation only one UK EQA scheme included participants using the Revvity PlGF method. Samples from this scheme were analysed by the Revvity method to confirm the performance of the PlGF method within the current study was consistent with laboratories already using the Revvity PlGF method.

### Statistics

Statistical analysis was performed using Analyse-IT® software. Continuous parametric data is presented as mean and standard deviation (SD) and non-Gaussian data as median and interquartile range (IQR). Spearman’s rank coefficient assessed the correlation between the Roche and Revvity methods using p < 0.05 as a statistically significant threshold. Passing-Bablok linear regression and Bland-Altman difference plots were used to assess agreement and relative bias following the Clinical Laboratory Standards Institute (CLSI) EP09-A3 guideline [23]. The 95% confidence intervals for the estimates of the slope and intercept associated with the Passing and Bablok regression are presented. To assess for concentration dependant relative bias data was partitioned into quartiles by mean concentration, and the relative bias in each quartile statistically evaluated by the Mann-Whitney test.

## Results

### Imprecision

Intermediate imprecision for the Revvity PlGF method expressed as percentage coefficient of variation was 9.0% at 29.6 pg/mL (n = 19) and 3.2% at 81.8 pg/mL (n = 18). Comparatively the Revvity sFlt-1 method had a coefficient of variation of 6.0% at 616.1 pg/mL (n = 19) and 3.4% at 4,477.0 pg/mL (n = 18).

**FIGURE 1:**
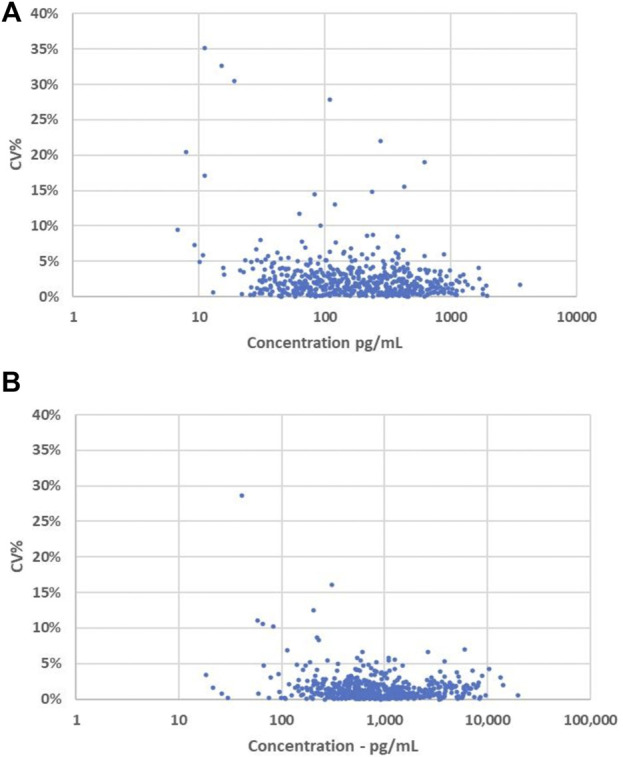
Revvity assay precision profiles derived from duplicate testing in clinical samples (n = 581) for **(A)** PlGF; **(B)** sFlt-1. The concentration (x-axis) is presented on a logarithmic scale as the results span the wide range observed in clinical practice.

Sample precision profiles, derived for both methods from 581 clinical samples analysed in duplicate are presented in Figure 1. For PlGF this covered a range of concentrations from <50 pg/mL to >1,000 pg/mL and for sFlt-1 covered a range of <10 pg/mL to >10,000 pg/mL. The median CV% for PlGF was 1.8%, IQR 2.3% and for sFlt-1 the median CV% was 1.1%, IQR 1.5%. PlGF imprecision at lower concentrations (<100 pg/mL, n = 195) was slightly higher at a median CV% of 1.9%, IQR 2.6% compared to higher results (>100 pg/mL, n = 386), median 1.5%, IQR 2.0%. Similarly, sFlt-1 imprecision at lower concentrations (<400 pg/mL, n = 135) was slightly higher, median CV% of 1.4%, IQR 1.7%, than at higher concentrations (>400 pg/mL, n = 446), median CV% of 1.0%, IQR of 1.4%.

### Comparability

**FIGURE 2:**
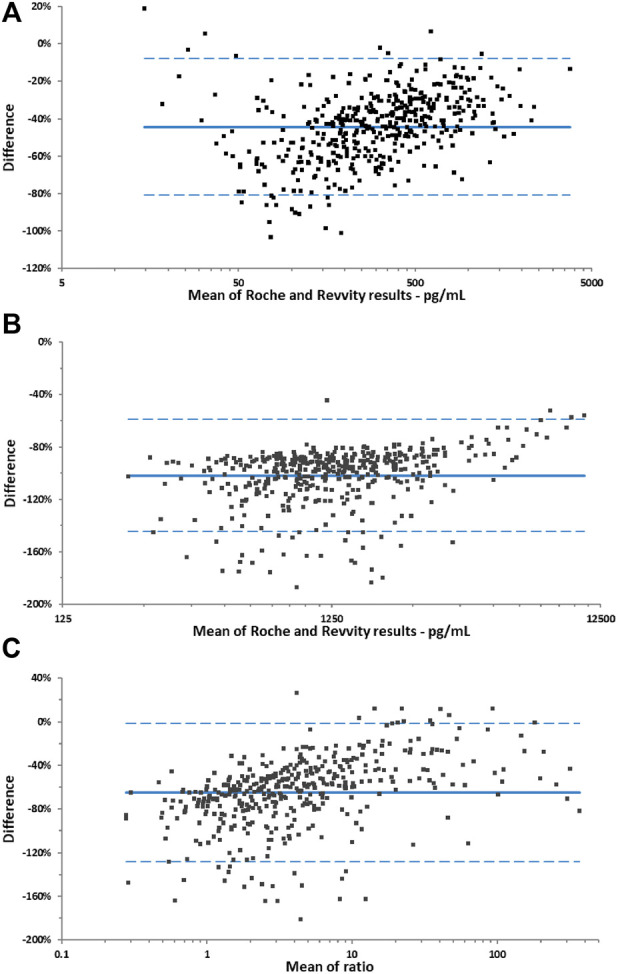
Bland-Altman plots showing percentage differences between the Revvity and the Roche methods in clinical sample (n = 437) for **(A)** PlGF, **(B)** sFlt-1 and **(C)** sFlt-1:PlGF ratio.

A total of 612 specimens (581 analysed in duplicate) were analysed for sFlt-1 and PlGF by both the Roche and Revvity methods. Following exclusions (repeat samples, twin pregnancies, and those tested at gestational ages outside the 2022 NICE guidance [14]) a total of 437 serum specimens were available to compare results between the two methods. The median PlGF concentration determined by the Roche method was 366.9 pg/mL, IQR 396.5, range 13.3–4,006 pg/mL compared to a median of 235.1 pg/mL, IQR 309.2, range 15.5–3506, when determined by the Revvity PlGF. Comparative data for the sFlt-1 assays were a median of 1810 pg/mL, IQR 1614.3, range 332–13,902, for the Roche method and a median of 577.1 pg/mL, IQR, 581.6, range 59.7–7829.4 for the Revvity method. Data is presented in Figure 2 as Bland-Altman plots which suggest a concentration dependant relative bias between the different manufacturer’s methods for both PlGF and sFlt-1 methods and the calculated sFlt-1:PlGF ratio.

**FIGURE 3:**
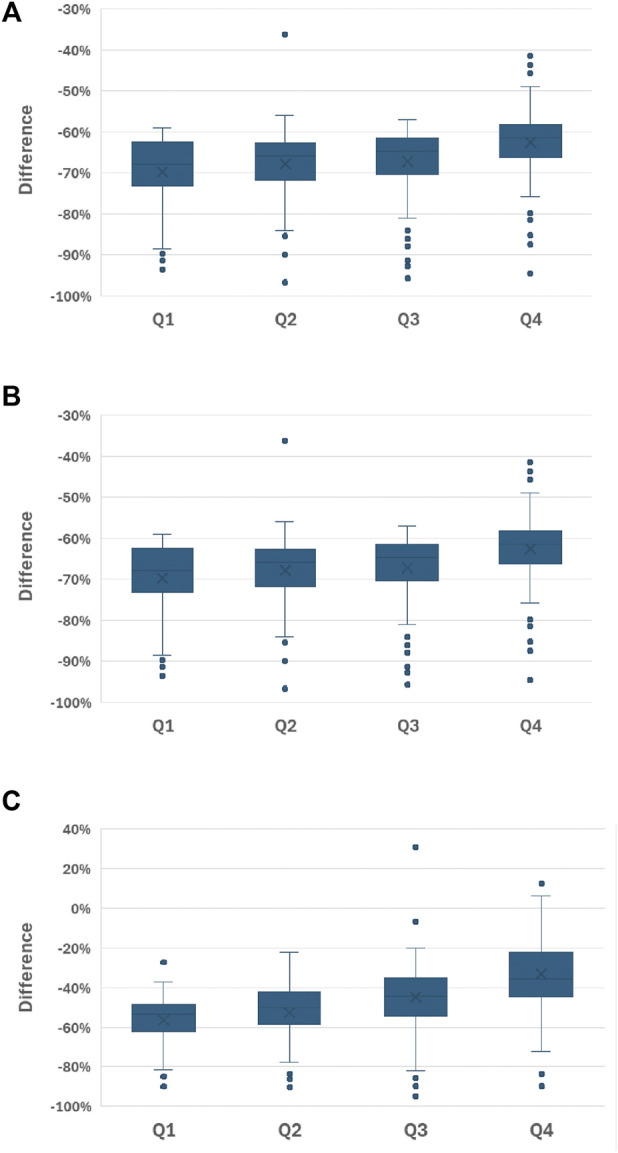
Comparative differences in bias relative to concentration, between Roche and Revvity methods presented as box as whisker plots by quartile for **(A)** PlGF, **(B)** sFlt-1 and **(C)** ratio.

Passing-Bablok regression between the methods demonstrated a relationship defined by the equations y = −23.4 + 0.73x (r = 0.983) for PlGF and y = −87.7 + 0.40x (r = 0.971), for sFlt-1. As the Bland-Altman plots suggest concentration dependant method relative bias the data was analysed further through Passing-Bablok regression undertaken on data subsets, partitioned by Revvity calibration concentrations (Table 1). Additionally, data was partitioned into quartiles (Q) by mean concentration (for PlGF and sFlt-1) or value (ratio) (Figure 3) and relative bias between each evaluated. The relative bias differences for PlGF were significant across all quartiles *(<0.001*) with the greatest significance being between Q1 and Q4 (*p < 0.0001*). For sFlt-1 the relative bias difference was significant between Q1 and Q4, and Q3 and Q4 (both *p < 0.0001*). For the sFl-1:PlGF ratio the relative bias differences between all quartiles were statistically significant being greatest (*p < 0.0001*) between Q3 and Q4 and least between Q1 and Q2 (*p < 0.01*).

**TABLE 1:** Passing-Bablok regression analysis evaluated by kit calibrant concentration range.

PlGF concentration range	Concentration range (pg/mL)	n	Intercept (95% CI)	Slope (95% CI)	Spearman rank coefficient (95% CI)
Results between calibrants A and C	<35.3	16	16.12 (11.45–28.88)	0.248 (0.0427–0.343)	0.644 (0.203–0.868)
Results between calibrants C and D	35.3 to 135	111	0.4635 (−7.947–9.296)	0.535 (0.483–0.597)	0.837 (0.769–0.887)
Results between calibrants D and E	135 to 700	260	−20.58 (−35.03 to −6.991)	0.7141 (0.678–0.752)	0.946 (0.931–0.958)
Results above calibrant E	>700	50	135.5 (−2.743–261.2)	0.6430 (0.524–0.749)	0.886 (0.804–0.935)
All data	15.5 to 3506	437	−23.39 (-30.37 to -18.36)	0.729 (0.711 to 0.749)	0.983 (0.979 to 0.986)
sFlt-1 concentration range					
Results between calibrants A and C	<134	13	n/a	n/a	−0.335 (−0.756 to 0.282)
Results between calibrants C and D	134 to 599	208	74.84 (30.99–125.9)	0.250 (0.207–0.296)	0.598 (0.500–0.681)
Results between calibrants D and E	599 to 3480	204	−27.71 (−76.12 to 30.83)	0.384 (0.356–0.405)	0.894 (0.861–0.919)
Results above calibrant E	>3480	12	−1,022 (−3366 to 164.5)	0.626 (0.482–0.822)	0.951 (0.824–0.987)
All data	0 to 3480	437	−87.67 (-113.4 to -62.62)	0.403 (0.388 to 0.416	0.884 (0.861 to 0.903)

**FIGURE 4:**
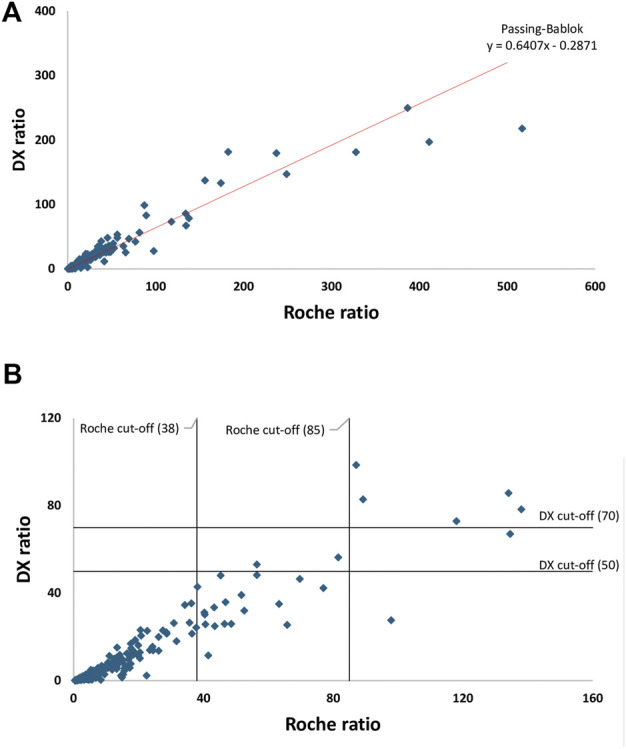
Comparability of the sFlt-1: PlGF ratio when determined using the Revvity and Roche methods (n = 437). **(A)** Passing and Bablok regression for all data. **(B)** Concordance of ratio results at lower values (below a ratio of 160 by the Roche methods) and relative to manufacturer specific decision thresholds.

The comparative sFlt-1:PlGF ratio from each of the instruments is presented in Figure 4. Passing- Bablok regression of the Roche ratio compared to the Revvity ratio showed a slope of 0.64 (confidence interval 0.61–0.67) and an intercept of −0.29 (confidence interval −0.39 to −0.23). Assessment of the concordance with respect to clinical categorisation against the manufacturer’s thresholds for rule-in and rule-out of PE are presented in Table 2 with overall agreement being 95.2% (n = 416), with 4.8% (n = 21) being non-concordant. Most (n = 18) of the specimens where non-concordant categorisation was observed had rule out results by the Revvity ratio and intermediate categorisation based on the Roche ratio. The majority (91.8%) of results obtained had ratios that were within the manufacturer specific rule out ranges for both methods.

**TABLE 2:** Categorical analysis: Concordance of sFlt-1:PlGF ratio results relative to manufacturer defined thresholds for rule in and rule out of preeclampsia.

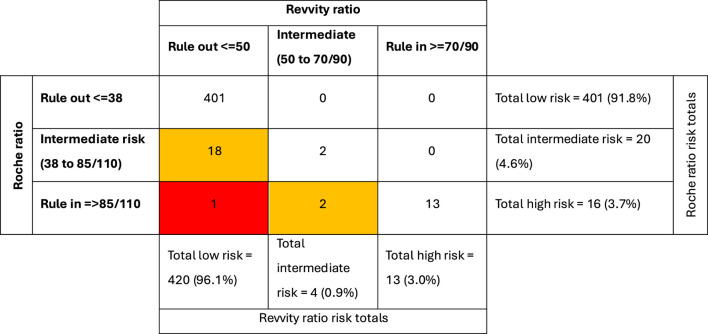

### Comparability of fresh vs. frozen stored sample

The majority (n = 550) of patient specimens analysed had been frozen at −80 °C following analysis using the Roche methods. To assess the potential impact of the freeze-thaw cycle on the relationship between the methods a further set of specimens (n = 62) were analysed by both methods without any freezing (Table 3). The Passing and Bablok regression slope and intercept for PlGF, sFlt-1 and the ratio had overlapping confidence intervals irrespective of whether the samples were fresh or frozen, and the categorisation against risk thresholds remained the same with either equation.

**TABLE 3:** Passing-Bablok regression equations for fresh and frozen data subsets.

​	n	sFlt-1	PlGF	Ratio
Fresh	62	y = −117.1 + 0.41x (r = 0.986)	y = −11.63 + 0.65x (r = 0.949)	y = −0.2974 + 0.645x (r = 0.974)
Frozen	550	y = −162 + 0.45x (r = 0.969)	y = −14.71 + 0.70x (r = 0.978)	y = −0.3862 + 0.667x (r = 0.933)

### External quality assessment samples

Comparable PlGF results were noted in six UKNEQAS samples against the Revvity group mean, results in the evaluation ranging from 19 to 67 pg/mL, mean 38 pg/mL, compared to target method means of 21–75 pg/mL, mean 40 pg/mL. The mean percentage difference was −4.5%, range 5.2% to −11.2% and mean absolute differences of 2 pg/mL, range one to −8 pg/mL. The Revvity results for the six EQA specimens were lower, being a mean of 68.7%, range 59.7%–81.4% of the schemes reported Roche results.

## Discussion

In this study we have evaluated the Revvity immunoassays for the analysis of PlGF and sFlt-1 using the DELFIA Xpress analyser. The methods were easy to set up and run specimens but as with other comparisons between immunoassays for these biomarkers [19, 20] there are notable concentration differences when measured with the two manufacturer’s methods. This confirms the importance of using method specific thresholds in clinical practice for both rule in and rule out PE criteria [12].

The intermediate imprecision of the Revvity methods for both PlGF and sFlt-1, is between 3.2 and 9.0 CV%, broadly similar to other automated methods [24]. This is demonstrated in both IQC materials provided by the manufacturer. It was also supported by precision profiles constructed from duplicate patient specimen analysis. Most paired duplicates had imprecision below 5 CV% across the concentration range studied for both assays and as with most immunoassays a trend towards higher imprecision at lower concentrations.

The correlation between the Roche and the Revvity methods for both PlGF and sFlt-1 show notable relative bias which appears to be concentration dependant. This is apparent in the Bland-Altman difference plots, the difference in slope value of the Passing-Bablok regression when analysed relative to the Revvity calibrant values, and the statistically different relative bias between most quartiles, for all measures. This had highest statistically significance between the lowest compared to highest quartiles.

A previous study comparing PlGF immunoassays in the context of first-trimester PE screening, with PlGF concentrations up to around 100 pg/mL, noted a regression slope of 0.553 (CI 0.518–0.588) between the Roche and Revvity (formerly PerkinElmer) methods [20]. This is consistent with the present study when the regression slope is considered at lower PlGF concentrations (up to 135 pg/mL) where the slope was 0.535 (CI 0.483–0.597).

The sFlt-1:PlGF ratio is used by both Roche and Revvity to support diagnosis of PE both as a rule in and rule out test. Categorisation against the manufacturers’ recommended thresholds demonstrates 95.2% concordance between the methods in this respect, with 4.8% of results not in agreement. Further evaluation with clinical follow up of patients is required to confirm clinical diagnostic accuracy and assess what factors contribute to this non-concordance. We recognise this as a limitation of the current study which focuses specifically on analytical performance.

A potential cause of disagreement between the methods is stability of PlGF and/or sFlt-1. There is data to support stability of both markers when measured by the Roche methods [24] but we are not aware of formal independent studies with respect to the Revvity method stability. In the present study both fresh and with once frozen samples were compared, and the consistent slope and intercept in these two groups would suggest stability is not a major contributor to the differences noted. We accept a limitation of our study is that we did not include a formal stability study of the Revvity method, and this should therefore be confirmed in the future.

The samples utilised in the study were those requested in clinical practice and in which we noted around 90% were within the low-risk category. This is a higher proportion than we observed in the INSPIRE study [10] and reported in other angiogenic marker studies in patients presenting with PE. Internal audits of test usage have shown that with time, greater test utilisation and that 90% of patients fall into the low-risk range. The reasons for this may include clinician familiarity and confidence in the tests but this requires further in-depth evaluation to confirm. The large number of samples (>600 samples) utilised in the comparison allowed the wide range of concentrations seen in clinical practice to be assessed and this mitigates the potential bias risk of the large proportion of samples with results in the low-risk category, however we recognise this may be a limitation of the study.

A further limitation of the study is that this was a pragmatic study within a clinical setting and whilst sample collection and arrival data indicate timely handling the time to centrifugation is assumed based on subsequent testing time on the instrument. It is possible some samples may not have been centrifuged immediately following arrival however this would be unusual due to streamlined workflows and clinical expectations.

The comparator PlGF and sFlt-1 methods in the present study are those from Roche which are widely used in the UK and in our laboratory have shown satisfactory performance in two EQA schemes. Whilst the Revvity PlGF method has been available for many years the Revvity sFlt-1 method and use in the ratio is relatively new. There are currently limited participants using Revvity methods in UK EQA schemes, but PlGF EQA results in the present study are consistent with other users of this method.

Immunoassays for peptides and proteins present unique measurement challenges including standardisation difficulties [25] when the measurand is not clearly defined, and antibody specificity varies between methods. For example, structural differences between forms of human chorionic gonadotrophin [26] have a significant impact on method agreement. Similarly, the development and calibration of comparable immunoassays for both free and total prostate specific antigen (PSA) was slow due to method dependant recognition of the complexed and free forms [27]. PlGF and sFlt-1 are also complex proteins and similar challenges may be noted as a greater number of methods are developed. PlGF and sFlt-1 both have isoforms that circulate, they bind to each other, and there are a wide range of structurally similar proteins which share protein sequences that could impact on any individual method response and the results obtained [28, 29]. Currently there is no agreed consensus on the measurand or common reference materials against which to calibrate PlGF and sFlt-1 which limits alignment of methods.

The PlGF test has been used as a standalone marker for PE and two manufacturers’ methods, the Triage method and the Revvity method have associated diagnostic criteria supported by NICE [14]. The relative merits of the sFlt-1:PlGF ratio compared to PlGF alone require further assessment in a large cohort of patients with clinical outcomes. A secondary analysis of the INSPIRE data set suggests that continuous values of sFlt-1 only or the sFlt-1/PIGF ratio may have better diagnostic accuracy compared to a PIGF only or the sFlt-1/PIGF ratio when used against defined thresholds [30]. Similar studies are required for all manufacturers methods to allow a fuller understanding of their relative clinical utility.

The consequence of the current non-quantitative equivalence is that results from different instruments, and the corresponding ratio require different clinical decision thresholds [14]. In several areas of obstetric practice, the use of multiples of the method median (MoMs) allows both comparability between methods and gestational age-related changes to be accounted for in the interpretation of results. A recent publication [31] explores the benefits of MoMs in the context of angiogenic markers for the diagnosis of PE and warrants further consideration although this will require method specific gestational age medians and regression relationships to be accurately determined.

The present study provides a large data set measuring both PlGF and sFLt-1 over the wide concentrations range observed in routine practice against NICE guidance HTG630. Notable differences are observed which impact on interpretation and variation in relative bias precludes interchangeability of results. Future investigations to identify the causes of these differences are warranted.

### Limitations

The comparisons included both fresh and frozen samples it is possible this may contribute to the relative differences observed but a sub-analysis within the results provides data that suggests this was not a major contributor to the relative differences observed.

## Summary table

### What is known about this subject

- Preeclampsia (PE) is a major clinical complication of pregnancy that in some cases may be challenging to diagnose with clinical assessment alone.
- The angiogenic markers sFlt-1 and PlGF have good diagnostic accuracy for detection of preeclampsia.
- There are a growing number of methods for these markers, but a complete understanding of their comparability is lacking.

### What this paper adds

- The Revvity methods for PlGF and sFlt-1 are precise methods that are easy to use in practice.
- In patients with suspected PE there is correlation between Roche and Revvity methods but not numeric agreement.
- The relative bias between methods is concentration dependant which may impact of categorical agreement for risk stratification of patients with suspected PE.

## Concluding statement

This work represents an advance in biomedical science because it demonstrates analytical differences between results of angiogenic biomarkers measured with different manufacturers methods used in clinical practice.

## Data Availability

The raw data supporting the conclusions of this article will be made available by the authors, without undue reservation.
